# Atrophy of the Vagus Nerve in Parkinson's Disease Revealed by High-Resolution Ultrasonography

**DOI:** 10.3389/fneur.2018.00805

**Published:** 2018-09-27

**Authors:** Uwe Walter, Panagiota Tsiberidou, Maxi Kersten, Alexander Storch, Matthias Löhle

**Affiliations:** ^1^Department of Neurology, University of Rostock, Rostock, Germany; ^2^German Centre for Neurodegenerative Diseases, Rostock/Greifswald, Rostock, Germany

**Keywords:** Parkinson's disease, vagus nerve, accessory nerve, phrenic nerve, ultrasound, non-motor symptoms

## Abstract

**Background:** The vagus nerve has been suggested to represent one major route of disease progression in Parkinson's disease (PD). Here, we examined whether patients with idiopathic PD exhibit an atrophy of the vagus nerve in comparison to age-matched controls.

**Methods:** In this cross-sectional study, performed between July 2017 and January 2018, we measured the caliber (cross-sectional area) of the mid-cervical vagus, accessory and phrenic nerves in 20 patients with PD (disease duration: 10.1 ± 7.4 years) and 61 (including 20 age-matched) controls using high-resolution ultrasonography. Ultrasonography and assessments of autonomic function were performed by blinded raters.

**Results:** Mean vagus nerve calibers were lower in patients with PD compared to age-matched controls (right: 0.64 ± 0.17 vs. 1.04 ± 0.20; left: 0.69 ± 0.18 vs. 0.87 ± 0.15 mm^2^; *p* < 0.001) while accessory and phrenic nerve calibers did not differ. In controls, age correlated negatively with calibers of the accessory and the phrenic nerve (each *p* ≤ 0.001), and trended to correlate with vagus nerve caliber (*p* = 0.023). In patients with PD and age-matched controls combined, the summed caliber of the right and left vagus nerves correlated with the burden of autonomic symptoms on the PD Non-Motor Symptoms Questionnaire (*r* = −0.46; *p* = 0.003). Moreover, the caliber of the right but not of the left vagus nerve correlated with the parasympathetic domain of heart rate variability (*r* = 0.58; *p* = 0.001).

**Conclusions:** PD is associated with a bilateral atrophy of the vagus nerve but not of the spinal accessory or the phrenic nerves. Our findings suggest that viscero-afferent and viscero-efferent vagal fibers are predominantly affected in PD.

## Introduction

The vagus nerve has been repeatedly suggested to represent one major route of disease progression in Parkinson's disease (PD), with an active retrograde transport of α-synuclein originating in the enteric nervous system ascending the vagus nerve and eventually reaching the dorsal motor nucleus of the vagus (dmX) in the lower brainstem (1). In support of this hypothesis, subdiaphragmatic truncal vagotomy has been reported to be associated with a decreased risk for subsequent PD (2, 3). In animal models, α-synuclein derived from human PD brain lysate and distinct recombinant α-synuclein forms are transported via the vagal nerve and reach the dmX in a time-dependent manner after injection into the intestinal wall (4). Axonal-predominant α-synuclein pathology has also been found in the glossopharyngeal-vagus and spinal nerve roots as well as in cervical and pharyngeal sections of the vagus nerve in PD patients (5, 6). However, it is still unknown whether the neurodegenerative process in PD is also associated with measurable atrophy of the vagus nerve *in vivo*.

High-resolution ultrasound (HR-US) is the method of choice for imaging mid-cervical cranial and spinal nerves (7–10). Mild to moderate atrophy of the vagus nerve has been detected on HR-US in amyotrophic lateral sclerosis and in diabetic neuropathy (7, 9). In order to investigate whether the vagus nerve exhibits sonographic signs of atrophy in PD, we measured the cross-sectional area (CSA) of the cervical vagus, accessory and phrenic nerve in PD patients and in control subjects.

## Methods

### Study sample

We recruited 81 adult participants between July 2017 and January 2018 at the Department of Neurology of the University of Rostock, Germany. Twenty participants had PD (disease duration: 10.1 ± 7.4 years, range 1–26 years; motor subtypes: 11 akinetic-rigid, 4 mixed, 3 tremor-dominant, 2 postural instability/gait difficulty), and 61 were control subjects (Table 1). The control group comprised healthy volunteers from our hospital staff (*n* = 52) and patients with minor stroke (*n* = 19) who had no history of diabetes, impaired glucose tolerance, severe hypertension, coronary heart disease, neurodegenerative disease or other relevant chronic disorders. None of the PD patients had diabetes, impaired glucose tolerance, severe hypertension, coronary heart disease, other neurodegenerative disorders, or relevant chronic disorders.

**Table 1:** Demographic, clinical, neurophysiologic, and ultrasound findings.

	**All controls (*N* = 61)**	**Age-matched controls^a^ (*N* = 20)**	**PD patients (*N* = 20)**	***P*^b^**
**DEMOGRAPHICS**				
Age (years)	45.1 ± 20.7	70.1 ± 10.2	73.2 ± 6.7	0.26^c^
Gender, female/male	38/23	10/10	7/13	0.52^d^
Height (cm)	173.8 ± 8.1	170.5 ± 8.2	168.7 ± 7.1	0.45^c^
Weight (kg)	75.1 ± 16.8	78.2 ± 18.2	74.9 ± 16.8	0.55^c^
PD duration (years)			10.1 ± 7.4	
**CLINICAL SCORES**				
MDS-UPDRS-III			30.7 ± 12.4	
PD-NMSQ	1.8 ± 2.2	3.2 ± 2.2	9.7 ± 5.6	<**0.001**^c^
**NEUROPHYSIOLOGY**				
RMSSD (ms)^e^		0.037 ± 0.037	0.050 ± 0.062	0.49^c^
**ULTRASONOGRAPHY (CSA, mm**^2^ **)**				
Right vagus nerve	1.32 ± 0.50	1.04 ± 0.20	0.64 ± 0.17	<**0.001**^c^
Left vagus nerve	1.12 ± 0.52	0.87 ± 0.15	0.69 ± 0.18	**0.001**^c^
Bilateral vagus nerve (sum)	2.44 ± 0.89	1.91 ± 0.38	1.33 ± 0.30	<**0.001**^c^
Right accessory nerve	0.46 ± 0.12	0.39 ± 0.12	0.43 ± 0.16	0.41^c^
Left accessory nerve	0.49 ± 0.16	0.40 ± 0.09	0.47 ± 0.15	0.10^c^
Bilateral accessory nerve	0.95 ± 0.23	0.80 ± 0.18	0.90 ± 0.28	0.18^c^
Right phrenic nerve	0.51 ± 0.13	0.42 ± 0.13	0.45 ± 0.15	0.56^c^
Left phrenic nerve	0.50 ± 0.13	0.44 ± 0.12	0.47 ± 0.12	0.53^c^
Bilateral phrenic nerve	1.01 ± 0.21	0.87 ± 0.23	0.92 ± 0.21	0.47^c^

Values except gender are mean ± SD. CSA, cross-sectional area in mm^2^; MDS-UPDRS-III, motor part of the MDS-sponsored Unified PD rating Scale; PD-NMSQ, PD Non-Motor Symptoms Questionnaire; RMSSD, root mean square of successive differences of R-R intervals on electrocardiogram with 0.2-Hz metronom-guided breathing.

a Group consisting of 20 control subjects age-matched to the group of PD patients selected from the entire control group.

b P-values are from comparisons between PD patients and 20 age-matched controls.

c t-test.

d Fisher's exact test.

e Validated for further analysis in 14 PD patients and 14 controls.

*Significant P-values in bold*.

### Clinical assessments

All participants underwent clinical, sonographic and neurophysiologic investigations on the same day. The severity of PD motor symptoms was evaluated with the Movement Disorder Society revised version of the Unified PD Rating Scale (MDS-UPDRS) part III (11). Non-motor symptoms were assessed using the validated German version of the PD Non-Motor Symptoms Questionnaire (PD-NMSQ) (12).

### Heart rate variability

To obtain standard time-domain heart rate variability parameters, we performed a short bedside analysis of electrocardiogram in the PD patients and age-matched control subjects. Following 5 min of inactivity in a supine position, 5-min resting 4-lead electrocardiogram (aV_R_, aV_L_, N, aV_F_) was recorded during daylight hours in a non-fasting state with 0.2-Hz metronom-guided breathing using a neurophysiologic workstation (Keypoint G4; Natus Europe GmbH, Planegg, Germany) (13).

R-R intervals were automatically detected and visually inspected; records with atrial fibrillation or ectopic beats were not used for the analysis. The root mean square of successive differences of R-R intervals (RMSSD) was calculated as a parameter reflecting parasympathetic (vagal) innervation (14).

### High-resolution ultrasonography

A high-end ultrasound system (MyLabTwice; Esaote SpA, Genova, Italy) equipped with a 15.0-MHz transducer (LA435) was applied for all sonographic measurements. PD patients and controls were investigated in random order in supine position. To ensure blinding with respect to the diagnosis of study participants, the ultrasound investigator (PT) was only allowed to see and study participants after they had already been seated on the investigation chair. Bilateral vagus, spinal accessory, and phrenic nerves were scanned in the axial view at the level of thyroid cartilage with the probe orientation marker directed toward the participant's anterior side as described earlier (8, 9). In order to obtain exact estimates of the CSA, we measured the longest cross-sectional diameter *a* and the (usually shorter) diameter *b* perpendicular to *a*, and calculated the elliptic CSA according to the formula: CSA = *a*·*b*·π/4. We used this approach since alternative methods reported previously (e.g., area tracing) had been reported to result in only rough measures of thin nerve CSA (7–10).

### Statistical analysis

Normally distributed variables were compared with the two-sided *t*-test, categorical variables were evaluated with Fisher's exact test. Spearman's rank correlation coefficients were calculated to correlate CSA measures with age, PD duration, MDS-UPDRS part III score, PD-NMSQ score and RMSSD; since five parameters were tested, we applied a Bonferroni correction for multiple comparisons, with *p* < 0.01 indicating significance. Statistical analyses were performed with SPSS software, version 22 (IBM Corporation, Chicago, IL).

## Results

**Figure 1:**
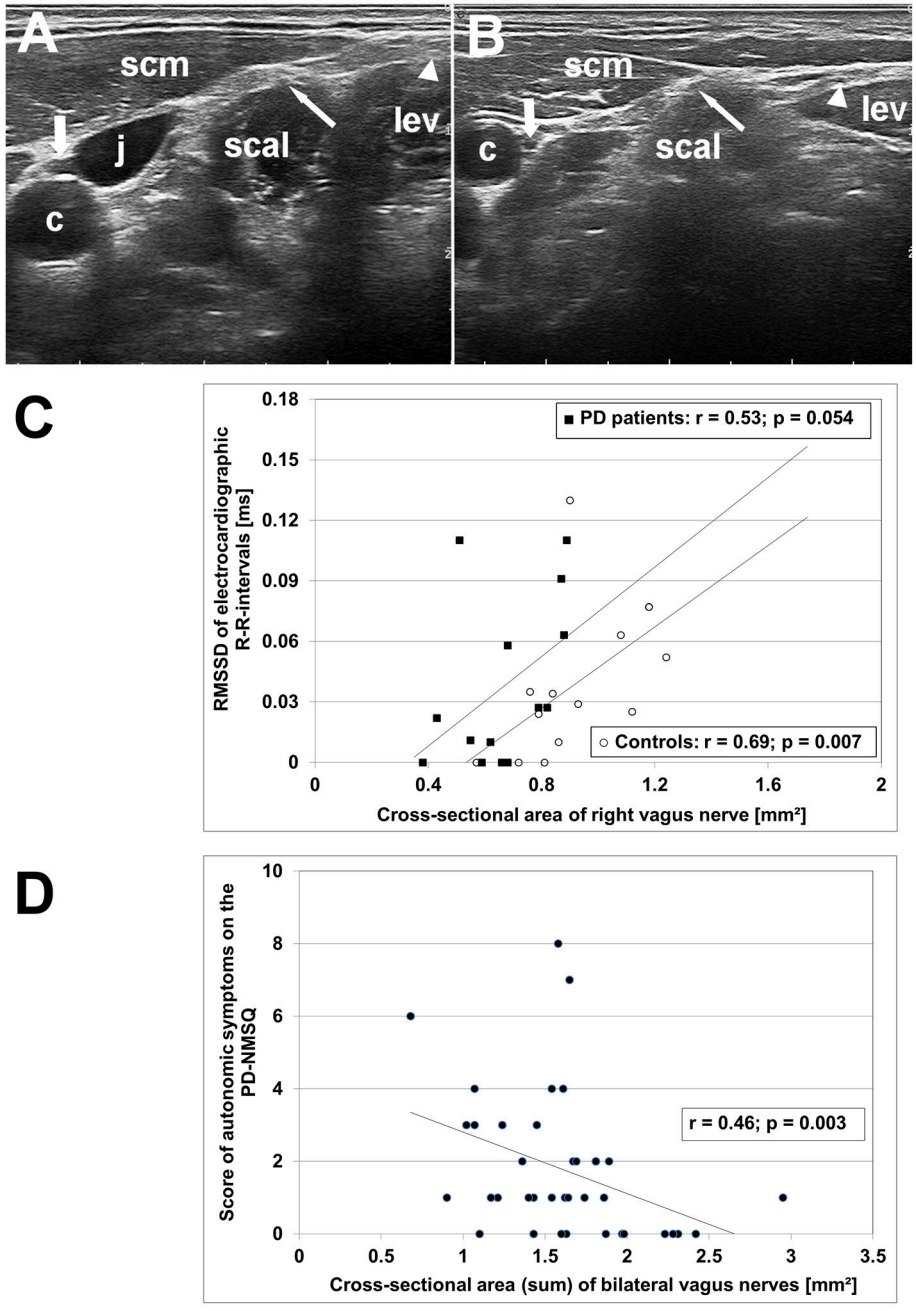
High-resolution ultrasonography (HR-US) findings of vagus, spinal accessory and phrenic nerves in PD patients and controls. **(A)** Axial HR-US scan of the lateral cervical region at the midneck level in a healthy elderly woman. The vagus nerve (thick arrow) is visualized in the carotid sheath between the common carotid artery (c) and the jugular vein (j). The phrenic nerve (thin arrow) is located superficial to the scalene muscle (scal) underneath the sternocleidomastoid muscle (scm). The spinal accessory nerve (triangle) is identified superficial to the levator scapulae muscle (lev). **(B)** Axial HR-US scan of the lateral cervical region in a PD patient. Compared to the control subject shown in **(A)** the vagus nerve (thick arrow) shows a clearly reduced caliber. The phrenic nerve (thin arrow) and the spinal accessory nerve (triangle) are of similar size as in age-matched controls. **(C)** Diagram showing the correlation between RMSSD, an electrocardiographic parameter reflecting vagal cardiac innervation, and caliber of right vagus nerve in PD patients (°) and age-matched controls (■). **(D)** Diagram showing the correlation between the sum score of autonomic symptoms on the PD Non-Motor Symptoms Questionnaire (calculated from items 1, 3, 4, 5, 6, 7, 8, 11, 19, 20, 28) and bilateral vagus nerve caliber in the combined group of PD patients and age-matched controls.

The clinical and ultrasonographic findings are summarized in Table 1. The CSA of right and left vagus nerve was significantly smaller in PD patients compared to age-matched controls (*p* < 0.001), whereas calibers of spinal accessory and phrenic nerves did not differ (Figure 1). In controls, vagus nerve CSA was larger on the right compared to the left side (1.32 ± 0.51 vs. 1.12 ± 0.53 mm^2^, *p* = 0.032). This asymmetry was not found in PD patients (0.64 ± 0.17 vs. 0.69 ± 0.18, *p* = 0.49). Patients and age-matched controls had similar accessory and phrenic nerve CSA. Right and left CSA of these nerves did not differ.

In controls, age correlated negatively with CSA of the accessory (*p* < 0.001) and the phrenic nerve (*p* = 0.001), and trended to correlate with vagus nerve CSA (*p* = 0.023). In patients, vagus nerve CSA did not correlate with disease duration, nor with cumulative levodopa dose (each, *p* > 0.2). Left vagus nerve CSA correlated with symptom severity on the MDS-UPDRS part III (*r* = 0.58; *p* = 0.007), unlike right vagus nerve CSA (*p* = 0.53). In the combined group of patients and age-matched controls, RMSSD correlated with the CSA of right (*r* = 0.58; *p* = 0.001) but not left vagus nerve (*p* = 0.24). Moreover, bilateral vagus nerve CSA correlated negatively with the PD-NMSQ total score (*r* = −0.51; *p* = 0.001) and with the sum score of autonomic items of the PD-NMSQ (*r* = −0.46; *p* = 0.003; Figure 1).

## Discussion

Our study demonstrates bilateral atrophy of the vagus nerve but not of the spinal accessory or the phrenic nerves in PD patients compared to age-matched controls. In controls, calibers of the spinal accessory and the phrenic nerves decreased with age, which to a lesser extent was also observed in the vagus nerve. The bilateral vagus nerve CSA correlated negatively with the burden of autonomic symptoms as measured with the PD-NMSQ. RMSSD, an electrocardiographic parameter reflecting vagal cardiac innervation, correlated with CSA of the right but not of the left vagus nerve.

A potential limitation of the present study is the limited number of patients. However the main finding of this study, i.e., the difference between vagus nerve calibers in PD patients vs. unrelated, age-matched controls, was statistically highly significant. To minimize the potential influences of co-morbidity known to be associated with vagal neuropathy (e.g., diabetes mellitus, severe coronary heart disease, chronic inflammatory neuropathies) we included only patients and controls who had no history and no diagnostic evidence from electrocardiogram or laboratory findings for any of these conditions. As we found no association of vagus nerve calibers with PD duration, nor with cumulative levodopa dose, it seems unlikely that vagus nerve atrophy was due to neuropathy discussed earlier to be associated with levodopa medication (15). The HR-US measures of vagus nerve CSA in our study were somewhat lower than values reported earlier for the normal population (7–10). This discrepancy may be explained by a more exact method applied in our study that involved estimation of both the long and short axis diameter in millimeters with two decimals, which were then used for calculating the elliptic CSA. However, our data are in line with previously reported long diameter measures (8). Despite considerable variability of vagus nerve CSA in both patients and controls, we found significant thinning of the vagus nerve in PD patients by about 30% on average compared to age-matched controls. This difference is more pronounced compared to previously reported mild thinning in amyotrophic lateral sclerosis (7). In contrast, patients with diabetic neuropathy were found to have similar or even more pronounced vagal atrophy (9). On the contrary, vagal hypertrophy has been observed in immune-mediated neuropathy (16).

In the cervical region, the vagus nerve contains unmyelinated viscero-sensory (including gustatory) fibers ascending mainly to the nucleus tractus solitarius (NTS), unmyelinated or partially myelinated viscero-motor and cardio-inhibitory fibers originating in the dmX, and thick myelinated somato-motor fibers originating in the nucleus ambiguous or from the cranial part of the accessory nerve. Neuronal degeneration with Lewy bodies and Lewy neurites in PD has been found to affect the dmX and the NTS, but not the nucleus ambiguous and the somatosensory nuclei (1, 17). The lack of atrophy in the solely somato-motor spinal accessory and phrenic nerves of PD patients in our study implies that myelinated somato-motor fibers are likely to be preserved in the vagus nerve as well. A recent study moreover showed normal somatosensory evoked potentials of the auricular branch of the vagus nerve in PD, suggesting absence of degeneration also in vagal fibers processing somatosensory information (18). The number of unmyelinated fibers is about four times higher than that of myelinated fibers in the vagus nerve (19, 20). If mainly affecting thin unmyelinated fibers, the extent of vagal atrophy of about 30% observed in our study would equal to a loss of approximately half of unmyelinated fibers in PD. Our finding that bilateral vagus nerve CSA correlates with the burden of autonomic symptoms also supports the view that vagal atrophy in PD mainly involves the unmyelinated fibers. Right vagus nerve CSA correlated with RMSSD, which is in line with the sinus node innervation by the right vagus (21).

It is important to note that our ultrasound study does not prove an ascending PD pathology from the gut via the vagus nerve, but may indicate a higher susceptibility of this long nerve to α-synucleinopathies. Thus, studies in larger samples of patients with PD and other forms of Parkinsonism are warranted to further elucidate the diagnostic value of vagus nerve HR-US.

## Ethics statement

The study was approved by the ethics committee at the Medical Faculty, University of Rostock (identifier: A 2017-0018). Written informed consent was obtained from each participant.

## Statistical testing

UW had full access to all the data in the study and takes responsibility for the integrity of the data and the accuracy of the data analysis.

## Author contributions

UW drafted and revised the manuscript for content, study concept, acquisition of data, analysis and interpretation of data, study supervision and coordination. PT, MK, and AS revising the manuscript for content, acquisition of data, analysis and interpretation of data. ML revising the manuscript for content, study concept, acquisition of data, analysis and interpretation of data.

### Conflict of interest statement

UW has received speaker honoraria and travel reimbursement from Merz Pharma, Bristol-Myers Squibb, Daiichi Sankyo, Bayer Vital, and Pfizer, and a research grant from Merz Pharma. He has received royalties from Kohlhammer Verlag and Elsevier Press. He serves as an editorial board member of Stem Cells, Stem Cells International, Open Biotechnology Journals, and jbc The Journal of Biological Chemistry. ML has received speaker honoraria from Bayer Vital. The remaining authors declare that the research was conducted in the absence of any commercial or financial relationships that could be construed as a potential conflict of interest.

## References

[1] Del Tredici K Braak H. Review: Sporadic Parkinson's disease: development and distribution of α-synuclein pathology. Neuropathol Appl Neurobiol. (2016) 42:33–50. 10.1111/nan.12298 26662475

[2] Svensson E Horváth-Puhó E Thomsen RW Djurhuus JC Pedersen L Borghammer P. Vagotomy and subsequent risk of Parkinson's disease. Ann Neurol. (2015) 78:522–9. 10.1002/ana.24448 26031848

[3] Liu B Fang F Pedersen NL Tillander A Ludvigsson JF Ekbom A. Vagotomy and Parkinson disease: a Swedish register-based matched-cohort study. Neurology (2017) 88:1996–2002. 10.1212/WNL.0000000000003961 28446653 PMC5440238

[4] Holmqvist S Chutna O Bousset L Aldrin-Kirk P Li W Björklund T. Direct evidence of Parkinson pathology spread from the gastrointestinal tract to the brain in rats. Acta Neuropathol. (2014) 128:805–20. 10.1007/s00401-014-1343-6 25296989

[5] Mu L Sobotka S Chen J Su H Sanders I Adler CH. Alpha-synuclein pathology and axonal degeneration of the peripheral motor nerves innervating pharyngeal muscles in Parkinson disease. J Neuropathol Exp Neurol. (2013) 72:119–29. 10.1097/NEN.0b013e3182801cde 23334595 PMC3552335

[6] Nakamura K Mori F Tanji K Miki Y Toyoshima Y Kakita A. α-Synuclein pathology in the cranial and spinal nerves in Lewy body disease. Neuropathology (2016) 36:262–9. 10.1111/neup.12269 26563477

[7] Grimm A Décard BF Athanasopoulou I Schweikert K Sinnreich M Axer H. Nerve ultrasound for differentiation between amyotrophic lateral sclerosis and multifocal motor neuropathy. J Neurol. (2015) 262:870–80. 10.1007/s00415-015-7648-0 25626722

[8] Hong MJ Baek JH Kim DY Ha EJ Choi WJ Choi YJ. Spinal accessory nerve: ultrasound findings and correlations with neck lymph node levels. Ultraschall Med. (2016) 37:487–91. 10.1055/s-0034-1385673 25520295

[9] Tawfik EA Walker FO Cartwright MS El-Hilaly RA. Diagnostic ultrasound of the vagus nerve in patients with diabetes. J Neuroimaging (2017) 27:589–93. 10.1111/jon.12452 28524416

[10] Pelz JO Belau E Henn P Hammer N Classen J Weise D. Sonographic evaluation of the vagus nerves: protocol, reference values, and side-to-side differences. Muscle Nerve (2018) 57:766–71. 10.1002/mus.25993 29053902

[11] Goetz CG Tilley BC Shaftman SR Stebbins GT Fahn S Martinez-Martin P. Movement Disorder Society-sponsored revision of the Unified Parkinson's Disease Rating Scale (MDS-UPDRS): scale presentation and clinimetric testing results. Mov Disord. (2008) 23:2129–70. 10.1002/mds.22340 19025984

[12] Storch A Odin P Trender-Gerhard I Fuchs G Reifschneider G Ray Chaudhuri K Non-motor Symptoms Questionnaire und Scale für das idiopathische Parkinson-Syndrom. Interkulturell adaptierte Versionen in deutscher Sprache. Nervenarzt (2010) 81:980–5. 10.1007/s00115-010-3010-z 20414634

[13] Gurevich TY Groozman GB Giladi N Drory VE Hausdorff JM Korczyn AD. R-R interval variation in Parkinson's disease and multiple system atrophy. Acta Neurol Scand. (2004) 109:276–9. 10.1111/j.1600-0404.2004.00226.x 15016010

[14] Task Force of the European Society of Cardiology and The North American Society of Pacing and Electrophysiology Heart rate variability. Standards of measurement, physiological interpretation, and clinical use. Circulation (1996) 93:1043–65. 10.1161/01.CIR.93.5.1043 8598068

[15] Nolano M Provitera V Manganelli F Iodice R Stancanelli A Caporaso G. Loss of cutaneous large and small fibers in naive and l-dopa-treated PD patients. Neurology (2017) 89:776–84. 10.1212/WNL.0000000000004274 28747449

[16] Grimm A Thomaser AL Peters N Fuhr P. Neurological picture. Vagal hypertrophy in immune-mediated neuropathy visualised with high-resolution ultrasound (HR-US). J Neurol Neurosurg Psychiatry (2015) 86:1277–8. 10.1136/jnnp-2014-308271 25209417

[17] Seidel K Mahlke J Siswanto S Krüger R Heinsen H Auburger G. The brainstem pathologies of Parkinson's disease and dementia with Lewy bodies. Brain Pathol. (2015) 25:121–35. 10.1111/bpa.12168 24995389 PMC4397912

[18] Weise D Adamidis M Pizzolato F Rumpf JJ Fricke C Classen J. Assessment of brainstem function with auricular branch of vagus nerve stimulation in Parkinson's disease. PLoS ONE (2015) 10:e0120786. 10.1371/journal.pone.0120786 25849807 PMC4388709

[19] Hoffman HH Schnitzlein HN. The numbers of nerve fibers in the vagus nerve of man. Anat Rec. (1961) 139:429–35. 13963923 10.1002/ar.1091390312

[20] Pereyra PM Zhang W Schmidt M Becker LE. Development of myelinated and unmyelinated fibers of human vagus nerve during the first year of life. J Neurol Sci. (1992) 110:107–13. 10.1016/0022-510X(92)90016-E 1506849

[21] Chen M Yu L Ouyang F Liu Q Wang Z Wang S The right side or left side of noninvasive transcutaneous vagus nerve stimulation: based on conventional wisdom or scientific evidence? Int J Cardiol. (2015) 187:44–5. 10.1016/j.ijcard.2015.03.351 25828310

